# Omnidirectional surface wave cloak using an isotropic homogeneous dielectric coating

**DOI:** 10.1038/srep30984

**Published:** 2016-08-05

**Authors:** R. C. Mitchell-Thomas, O. Quevedo-Teruel, J. R. Sambles, A. P. Hibbins

**Affiliations:** 1 Department of Physics and Astronomy, University of Exeter, UK; 2 School of Electrical Engineering, KTH Royal Institute of Technology, Sweden

## Abstract

The field of transformation optics owes a lot of its fame to the concept of cloaking. While some experimental progress has been made towards free-space cloaking in three dimensions, the material properties required are inherently extremely difficult to achieve. The approximations that then have to be made to allow fabrication produce unsatisfactory device performance. In contrast, when surface wave systems are the focus, it has been shown that a route distinct from those used to design free-space cloaks can be taken. This results in very simple solutions that take advantage of the ability to incorporate surface curvature. Here, we provide a demonstration in the microwave regime of cloaking a bump in a surface. The distortion of the shape of the surface wave fronts due to the curvature is corrected with a suitable refractive index profile. The surface wave cloak is fabricated from a metallic backed homogeneous dielectric waveguide of varying thickness, and exhibits omnidirectional operation.

The creation of an efficient and precise cloak has been the ambition of engineers for decades^1^,^2^. Additionally, the use of low profile technology is highly advantageous in the pursuit of miniaturisation of integrated electromagnetic devices. These two aims have prompted the recent interest in surface wave cloaks. Based on the advent of transformation optics, researchers have explored a number of experimental implementations of surface wave cloaks^3^,^4^,^5^. These design approaches can broadly be separated into two categories: Planar cloaks and volumetric cloaks. In planar cloaks, the material properties are engineered to guide the waves around a certain area^4^,^5^,^6^ that is then free from surface wave scattering. For surface waves, the most well-known cloak design^7^ cannot be implemented because of the high degree of anisotropy and sub-unity index values. However, it is possible to employ a planar carpet cloak design to create a cloaked area^4^,^5^,^6^. This necessarily implies that the surface cloak is restricted to unidirectional operation^4^ because the transformation takes a finite area on the surface and makes it appear as if it were a line segment to waves propagating on the surface. Therefore, only when the waves are propagating in a direction perfectly parallel to the line will the cloak effectively eliminate any scattering. Another planar transformation approach has also been developed specifically for two dimensional systems^8^ and although this may provide omnidirectional solutions, once again the main drawback is the necessity of anisotropy.

Alternatively, volumetric carpet cloaks have also been investigated for surface waves^2^,^5^,^9^. These three-dimensional cloaks are necessarily larger than the cloaked region due to the requirement to satisfy the quasi-conformal approximation^10^. This problematically results in a cloak with large dimensions. The material properties required for these carpet cloaks can either be spatially varying and isotropic^10^, or homogeneous and anisotropic^11^. However, the former option has the disadvantage of degradation in performance due to the approximations that must be applied to ensure isotropy^12^. The latter uses resonant elements to obtain the degree of anisotropy required and is therefore severely bandwidth limited^11^.

In this work, rather than build upon the cloaks originally designed for free space waves, the approach taken here dispenses with the transformation optics technique altogether. Instead, a geometrical optics route is taken so that surface curvature can be beneficially employed^13^,^14^. While there is no analogy in three dimensions, for surface wave devices, the curvature of the surface becomes an extra degree of freedom which can be included into the cloak design process. This has been theoretically shown in previous work^15^, where it was demonstrated that a cloak can be designed by making a curved two dimensional surface appear as if it were flat to rays that are confined to it. As such, this method is able to cloak any object that is positioned underneath the curved portion of the guide, so that the wavefront shape of the surface wave will bear no signature of either the curvature of the guide or the object itself. While adding curvature to a surface wave system will inevitably lead to a degree of radiation from the surface^16^, this approach has the advantage of creating cloaks that are omnidirectional, isotropic and thin; both with respect to the dimension of the object being cloaked and the wavelength of operation.

## Results

**Figure 1:**
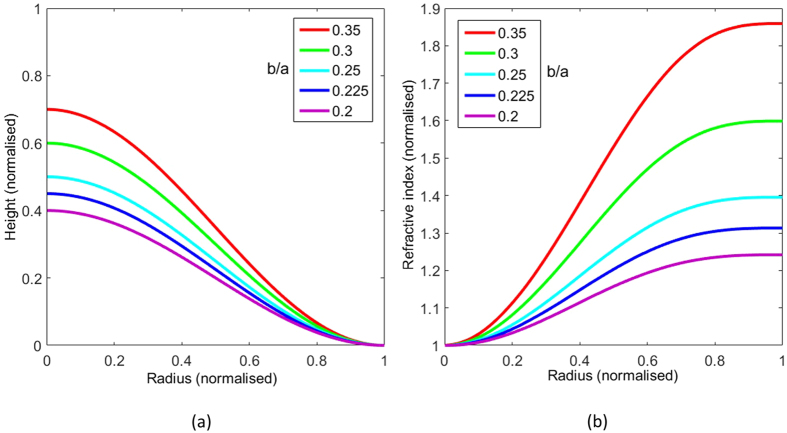
Design of the cloak. (**a**) Cross section of a cosine shaped obstacle where the height of the surface is given by *z* = *b*cos(*rπ*/*a*) + *b*. (**b**) Normalised mode index requirement for these cosine shaped obstacles to appear flat.

As discussed in ref. 15, our approach to surface wave cloaking can be applied to any rotationally symmetric surface deformation. For our experimental study, we have chosen to create the cloak from a cosine function. This was selected because it will attach in a continuous manner to a flat surrounding plane, thus preventing any reflections at this boundary. Figure 1(a) shows the cross-section of a selection of cosine function given by z = *b*cos(*rπ*/*a*) + *b* with different maximum height to radius ratios (*b/a*). Figure 1(b) shows the mode-index profiles necessary to create the illusion of a flat homogeneous surface by maintaining the same optical path length for all rays, normalised so the minimum value is unity. These indices were numerically calculated using equation (1) taken from ref. 15


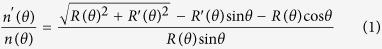


where *n*(*θ*) is the index we wish to find, *n′*(*θ*) is its derivative, *θ* is the angle between a position on the curved surface and the z-axis, and *R*(*θ*) is the associated length between the position and the origin, and *R*(*θ*)′ is its derivative.

There are a number of fabrication choices available to implement this variation in mode index, including a metasurface method with graded geometry^17^,^18^, or using dielectric layers, which can be employed together or independently to support and control surface wave propagation. Metasurfaces show great promise in their ability to create strong confinement of the surface wave and to permit a gradual variation of the mode index by changing the dimensions of each individual unit cell^19^,^20^. However, arranging these elements on a curved surface is problematic, as it is no longer possible to create a uniform unit cell size and shape across the surface. They also normally owe their behaviour to a resonant response, and are inherently narrow band.

In this work, the cloak was fabricated from a varying thickness homogeneous dielectric coating on a metal surface^21^. This benefits from simplicity of manufacture and the ability to achieve a continuous grading of the mode index. However, due to a weak confinement of the wave, it will suffer from higher radiation losses due to the curvature when compared to a metasurface implementation. By taking into account the maximum achievable mode-index contrast with a common dielectric medium (Perspex) with a relative dielectric constant of *ε*_*r*_ = 2.6 + 0.04i, the parameters given for the *b*/*a* = 0.225 profile (Fig. 1) were chosen for the test sample.

**Figure 2:**
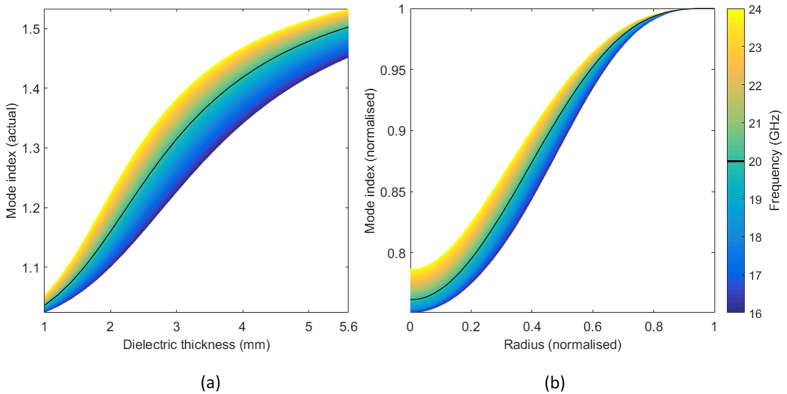
Frequency dependence of the cloak. (**a**) the mode index achieved by varying the dielectric coating thickness, for a range of frequencies, where the dielectric constant is *ε*_*r*_ = 2.6 + 0.04i. (**b**) variation of the mode index of the chosen cloak design with frequency. The black line highlights the design frequency at 20 GHz.

The bandwidth of such a device is limited due to the frequency dependence of the mode index. This layer design resembles an open dielectric waveguide, and as such, we expect its bandwidth to be much broader than that of a design consisting of resonant metamaterial elements. The frequency dependence of a varying thickness dielectric layer is given in Fig. 2(a) where it can be seen that the mode index of the lowest order TM mode only weakly depends on the frequency. In Fig. 2(b) the deviation from the required mode index of this cloak (black line) is shown. The mode index for each individual frequency is normalised to the corresponding maximum value in Fig. 2(a). This maximum value is the mode index in the external flat surface. At the centre of the cloak (*r* = 0) the mode index has only increased 3.3% at 24 GHz to the ideal cloak profile (black line in Fig. 2(b)); and reduced by 1.4% at 16 GHz. This illustrates the broadband performance of the cloak designed here.

## Simulated results

**Figure 3:**
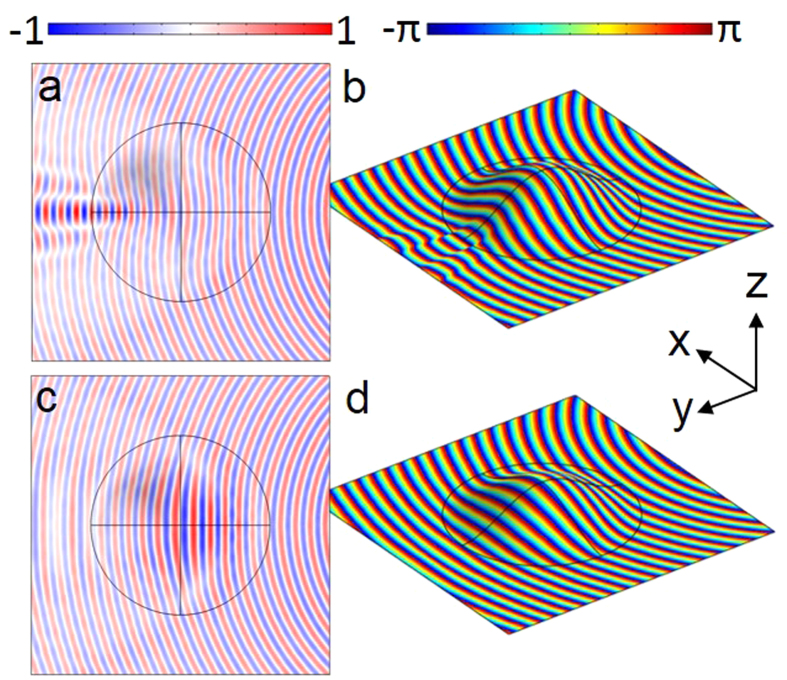
Simulation results. (**a,c**) the amplitude of the z-component of the electric field distribution at 20 GHz viewed in the x-y plane. (**b,d**) the phase data plotted from the same scan area. (**a,b**) are for a constant thickness sample, and (**c,d**) are for the cloak. All data is plotted at the upper surface of the dielectric.

Figure 3 shows predictions of the electric field distribution at the upper surface of the dielectric of both the uncloaked (constant thickness) and cloaked sample. The constant thickness sample is provided as a comparison because it realizes a homogeneous mode index to show the influence of the curvature on the propagation of surface waves. As can be seen in Fig. 3(a,b), the effect is to significantly distort the circular wavefront nature due to the varying optical path lengths over different parts of the curved guide. This creates a regions of destructive and constructive interference, clearly visible in the disturbance of the phase fronts. The model results from the cloak are shown in Fig. 3(c,d), where the circular nature of the surface wave emitted from a monopole source is clearly evident after the wave has passed over the curved portion of the surface. The consequence of this is that the curvature of the surface leaves little signature on the phase fronts of the surface wave, and the void created by the metal backed curved guide can be used to house any object beneath, rendering it undetectable.

## Measured data

**Figure 4:**
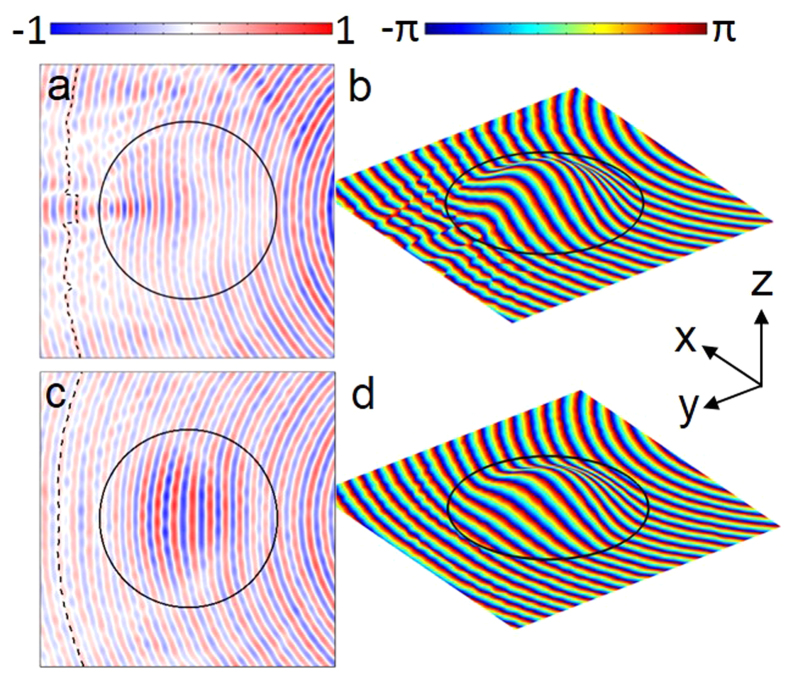
Measurement data. (**a,c**) the amplitude of the electric field distribution at 20 GHz detected by the near field probe viewed in the x-y plane. (**b,d**) the phase data plotted from the same scan area. (**a,b**) are for a constant thickness sample, and (**c,d**) are for the cloak. All data is taken from a distance 0.5 mm from the upper surfaces of the dielectric. Black dashed lines in **a** and **c** are lines of constant phase.

**Figure 5:**
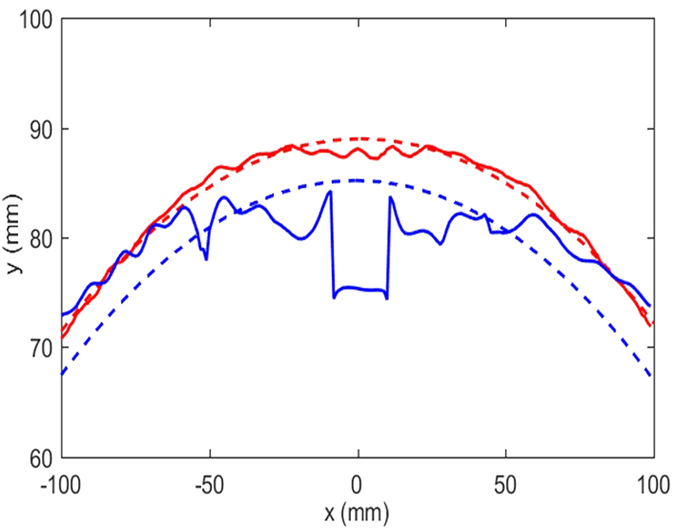
Lines of constant phase. Two example lines of constant phase for the constant thickness sample (solid blue) and the cloak (solid red). The dashed lines are circles fitted to each data set.

The plots in Fig. 4 display the raw measured data, again for both the cloak and the constant thickness sample. It can be seen that the measured data closely accords with the simulated data, demonstrating that accurate fabrication has been achieved. The higher amplitude of the signal in the centre of the cloak in Fig. 4(c) is caused by the fact that the mode index is lower in that region, so the mode is less confined. This is also mirrored in the simulated data. The dashed black line illustrates the position of the data given in Fig. 5.

Figure 5 displays a line of constant phase for each of the samples, the position of which is shown in Fig. 4, along with an arc fitted to each data set. Here, the arc represents the ideal case of an unperturbed cylindrical wave emitted from the fixed source position. To create this plot, the position was calculated at which the measured emitted wave has a given phase. For the constant thickness guide, the phase in the centre is retarded significantly with respect to the parts of the surface wave less affected by the curvature, evidenced by the trough in the solid blue curve in Fig. 5. The data from the cloak sample (solid red curve) shows excellent agreement with the predicted wavefront shape (dashed red curve). To quantify the performance of the cloak, the root mean squared (RMS) error of the measured phase to the fitted line is calculated. For the cloak, the RMS error is 0.77, while for the constant thickness guide, the value is 4.5, illustrating that the cloak has significantly restored the expected circular nature of the phase fronts emitting from the source antenna.

## Discussion

In conclusion, this work has experimentally demonstrated a surface wave cloak that utilises surface curvature to circumvent the requirement of extreme material properties. An omnidirectional and electrically thin surface wave cloak has been validated with the use of a homogeneous dielectric. The required mode index profile is achieved by a variation in the thickness of a homogeneous dielectric coating. This type of cloak design can be used in surface wave antenna applications where the need for conformality to an existing surface is vital, or where reduction of scattering from surface imperfections, which would otherwise have a detrimental effect on the antenna performance, is highly desirable.

## Methods

Both samples were fabricated using CNC milling, and are composed of Perspex with a permittivity of 2.6 + 0.04i at 20 GHz. The curved portion of the cloak varies from a thickness of 5.6 mm at the outer edge to 1.2 mm at the centre, and is backed with metallic foil. A mode index contrast of 1.31 at 20 GHz is obtained with this configuration for the lowest order TM mode. Both the cloak and the constant thickness sample have a radius of 60 mm (6 *λ*_*g*_, where *λ*_*g*_ is the wavelength in the waveguide), and are placed into a hole in a flat Perspex sheet of thickness 5.6 mm (approx. *λ*_*g*_/2). This sheet has tapered edges to ensure any surface waves incident on the edge of the sheet will radiate into free space to minimise reflections back into the scan area. The surface mode is excited with a coaxial antenna with 4.85 mm of the inner conductor exposed, and inserted into the dielectric layer from the underside, 198 mm away from the centre of the cloak. The coaxial probe antenna has a 0.5 mm exposed length, and is scanned at a distance of 0.5 mm from the surface of the dielectric to enable the imaging the surface wave. The scan area is 200 mm × 200 mm (20 *λ*_*g*_ × 20 *λ*_*g*_) and the probe is programmed to follow the surface of each sample, and the magnitude and phase data is recorded at 1 mm (0.1 *λ*_*g*_) intervals in the x-y plane.

### Data Availability

All experimental data used in this manuscript are openly available from the University of Exeter’s institutional repository at https://ore.exeter.ac.uk/repository/handle/10871/22667.

## Additional Information

**How to cite this article**: Mitchell-Thomas, R. C. *et al.* Omnidirectional surface wave cloak using an isotropic homogeneous dielectric coating. *Sci. Rep.* **6**, 30984; doi: 10.1038/srep30984 (2016).
